# Effects of *Chaenomeles japonica* Fruit Juice on Energy Balance and Biochemical and Histological Parameters in a Model of Diet-Induced Metabolic Syndrome in Rats

**DOI:** 10.3390/ph19040609

**Published:** 2026-04-10

**Authors:** Klementina Moneva-Marinova, Silvia Gancheva, Miroslav Eftimov, Maria Tzaneva, Milena Todorova, Mehmed Reyzov, Elis Rafailova, Maria Zhelyazkova-Savova, Stefka Valcheva-Kuzmanova

**Affiliations:** 1 Department of Pharmacology and Clinical Pharmacology and Therapeutics, Faculty of Medicine, Medical University “Prof. Dr. Paraskev Stoyanov”, Marin Drinov 55, 9002 Varna, Bulgaria; klementina.moneva@mu-varna.bg (K.M.-M.); silvia.gancheva@mu-varna.bg (S.G.); miroslav.eftimov@mu-varna.bg (M.E.); milena.todorova@mu-varna.bg (M.T.); mehmed.abtulov@mu-varna.bg (M.R.); elis.rafailova@mu-varna.bg (E.R.); zhelyazkova@mu-varna.bg (M.Z.-S.); 2 Department of Basic and Clinical Pathology, Forensic Medicine and Deontology, Faculty of Medicine, Medical University “Prof. Dr. Paraskev Stoyanov”, Hristo Smirnenski 1, 9010 Varna, Bulgaria; docent.tzaneva@gmail.com

**Keywords:** *Chaenomeles japonica*, Japanese quince, metabolic syndrome, oxidative status, visceral adiposity, rats

## Abstract

**Background/Objectives**: Metabolic syndrome (MS) is associated with an increased cardiovascular risk. The aim of this study was to reveal the effects of *Chaenomeles japonica* fruit juice (CJFJ) on energy balance and biochemical and histological parameters in rats with diet-induced MS. **Methods**: Fifty Wistar rats were allocated into five groups. For ten weeks, the Control group received a standard laboratory diet and tap water, while the other groups were given a high-fat high-fructose (HFHF) diet. The Control and MS groups were treated with distilled water, while the other three groups were treated with CJFJ at increasing doses. **Results**: Rats on an HFHF diet consumed less food and more liquids and had a higher caloric intake than the Control group. Among the CJFJ-treated animals, an increased food consumption, as well as an increased total caloric intake, and no difference in body weight gain were observed in comparison with the MS group. CJFJ did not affect glucose tolerance or the triglyceride and total cholesterol levels. CJFJ prevented an HFHF-induced decrease in superoxide dismutase and caused a decrease in thiobarbituric acid-reactive substances in serum. The medium CJFJ dose prevented an HFHF-induced increase in adipose tissue indices. Liver and adipose tissue histology revealed a protective effect of CJFJ. **Conclusions**: CJFJ may exert beneficial effects on visceral adiposity, oxidative status, and histopathological changes in the liver and adipose tissue in rats with diet-induced MS.

## 1. Introduction

Metabolic syndrome (MS) encompasses a cluster of metabolic disturbances, including insulin resistance, visceral obesity, hypertension and dyslipidemia, that increase the risk of cardiovascular disease and diabetes type 2, among others. Unhealthy nutritional patterns are often a major underlying cause. Insulin resistance, chronic low-grade inflammation and oxidative stress are some of the fundamental drivers of and contributors to the pathogenesis of MS. One of the earliest sources of this inflammation, also regarded as “metainflammation” or metabolic-induced inflammation, is suspected to be adipocyte hypertrophy and hyperplasia. This facilitates the occurrence of hypoxia in adipocytes far away from blood vessels, followed by necrosis, phagocyte accumulation and local inflammation that is intended to dispose of damaged cells [1]. Oxidative stress is generated, which drives the inflammatory process further, brings about inflammatory cytokine production and leads to cellular injury. Over time, pathological mechanisms unfold in various directions, engaging multiple pathways associated with MS. Despite its increasing prevalence and decreasing age of onset, limited advances have been achieved towards its prevention and management.

While dietary interventions remain one of the commonest approaches to MS, whether fruit juice (FJ) has a justifiable place as a part of them is highly controversial. While a valid source of vitamins, minerals and bioactive substances such as polyphenols, FJ is also a source of free sugars that can easily outweigh the health benefits, especially in the higher dose range. When investigating the relation of FJ with MS and with each of its components on its own, the results are highly variable based on the study design, context, etc. Part of the discrepancies arise from the failure to differentiate between FJ with added sugar and 100% FJ, with the evidence showing that their influence on metabolic outcomes is quite distinct [2]. Another discrepancy comes from the difficulty of properly estimating the exact amount of FJ consumed daily. D’Elia et al. reported an inverse relationship between 100% FJ consumption and cardiovascular risk—in particular, the risk of stroke [3]. In a prospective cohort study including 34,560 participants, Scheffers et al. found that consumption of up to seven glasses of pure FJ per week was associated with decreased risk of cardiovascular disease, while this benefit was lost with consumption of eight or more glasses weekly [4]. What is more, consumption of up to eight glasses of pure FJ weekly was also associated with a lower risk of stroke. The intake of 100% FJ was also associated with a decreased rate of anxiety in adults [5]. A meta-analysis of prospective studies including 49,591 participants reported a U-shaped dose–response association between 100% FJ consumption and MS, with protection at moderate doses [6]. It has been proposed that the inverse association between moderate FJ intake and MS is due to the nutritional content, while at higher amounts, the damaging properties of excessive sugar outweigh the benefits [7]. Recommendations for FJ intake vary greatly among countries [4,8,9] due to the mixed and inconclusive data.

The abundance of artificially sweetened beverages on the market and their increasing consumption draws attention to the high consumer interest in similar drinks, while also causing disappointment, since they are actually associated with increased risk of obesity [10], even though they provide fewer calories than the sugar-sweetened ones. So it seems like the quest for an alternative continues. Even though whole fruit consumption is generally regarded as the better option than consumption of FJ, juice might offer some advantages such as better nutrient bioavailability [8].

Plants used in traditional Eastern medicine are a focus of attention in the context of metabolic diseases. The genus *Chaenomeles*, belonging to the Rosaceae family, consists primarily of five species: *Chaenomeles speciosa*, *Chaenomeles thibetica*, *Chaenomeles cathayensis*, *Chaenomeles japonica*, and *Chaenomeles sinensis* [11]. While originating in East Asia, the plants have been introduced to all the continents on Earth except for Antarctica [12]. In vitro studies have reported antioxidant, lipid- and glucose-regulating, hepatoprotective, antineoplastic and antimicrobial effects of *Chaenomeles* [12]. The relatively low fructose content of *Chaenomeles* fruit juice in combination with the high concentration of polyphenolic compounds makes it a good candidate for use in obesity-related disorders. Fruit extracts from different *Chaenomeles* species have shown protective qualities in experimental models of diabetes mellitus [13], atherosclerosis [14], hyperuricemia [15], and depression [16]. *Chaenomeles japonica* (Thunb.) Lindl, also known as Japanese quince, is a species with many potential health benefits, few of which have been thoroughly studied.

Despite the growing body of evidence, the majority of studies on the genus *Chaenomeles* have focused on extracts or isolated bioactive compounds, while research using whole fruit juice remains limited. This distinction is important, as whole fruit juice preserves the natural composition of bioactive compounds and allows for potential synergistic interactions that may not occur when using isolated compounds or processed derivatives. Consequently, the biological effects of whole fruit juice may differ substantially from those reported for extracts. In this context, studying *Chaenomeles japonica* fruit juice (CJFJ) as a minimally processed, nutritionally relevant product may provide a more translationally meaningful insight into its potential role in metabolic disorders.

The aim of the present study was to evaluate the effects of CJFJ on energy balance and biochemical and histological parameters in rats with diet-induced MS, thereby expanding the currently limited evidence on the effects of whole-fruit-based interventions in a nutritionally relevant context.

## 2. Results

### 2.1. Effects of Chaenomeles japonica Fruit Juice on Energy Balance

**Figure 1:**
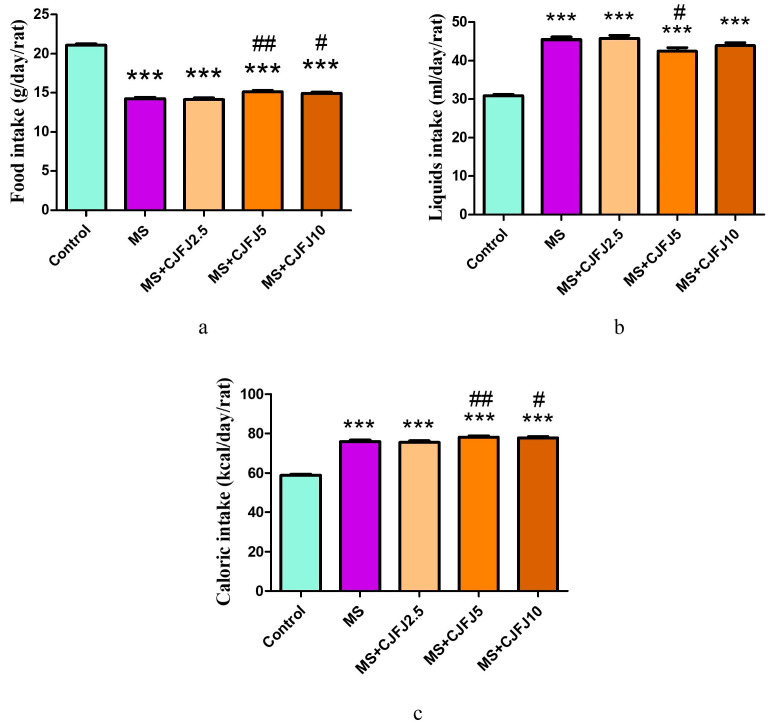
Food (**a**), liquid (**b**) and caloric (**c**) intake in rats with diet-induced metabolic syndrome (MS) treated with *Chaenomeles japonica* fruit juice (CJFJ) at doses of 2.5 mL/kg, 5 mL/kg and 10 mL/kg. *** *p* < 0.001 compared to Control group, ## *p* < 0.01, # *p* < 0.05 compared to MS group.

Parameters related to the energy balance are presented in Figure 1. Animals from the groups that were on a high-fat high-fructose (HFHF) diet consumed a smaller amount of food compared to the Control group (*p* < 0.001). Furthermore, a difference between the MS group and the treated groups (*p* < 0.001) was observed—CJFJ at doses of 5 mL/kg and 10 mL/kg increased the amount of food consumed.

The consumption of the 10% fructose solution by the groups on the HFHF diet was significantly higher (*p* < 0.001) compared to the consumption of tap water by the Control group. The analysis also revealed a difference between the MS group and the MS + CJFJ5 group—CJFJ at the dose of 5 mL/kg reduced the liquid intake (*p* < 0.05).

The total caloric intake in all groups on the HFHF diet was higher (*p* < 0.001) than that in the Control group. CJFJ at doses of 5 mL/kg and 10 mL/kg increased (*p* < 0.001) the caloric intake in comparison to the MS group.

### 2.2. Effects of Chaenomeles japonica Fruit Juice on Total Body Weight

**Figure 2:**
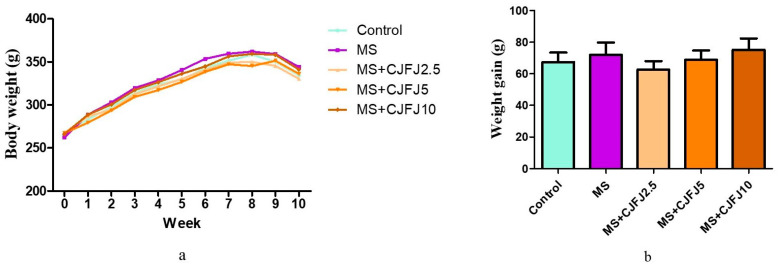
Body weight (**a**) and mean body weight gain (**b**) during the 10 weeks of the experiment in rats with diet-induced metabolic syndrome (MS) treated with *Chaenomeles japonica* fruit juice (CJFJ) at doses of 2.5 mL/kg, 5 mL/kg and 10 mL/kg.

The body weights throughout the experiment are shown in Figure 2a. Table 1 presents the body weights of the animals at the beginning and at the end of the experiment. The mean body weight gain did not differ among the animals from all experimental groups (Figure 2b). What is more, as depicted in Figure 2a, the body weight gain in all HFHF-diet groups followed the same variation pattern as the Control group throughout the entire experiment.

### 2.3. Effects of Chaenomeles japonica Fruit Juice on Glucose Tolerance Test (GTT)

The results from the GTT are presented in Table 2. The fasting blood glucose levels did not differ significantly among the experimental groups (4.46 ± 0.09 in Control, 4.48 ± 0.14 in MS, 4.44 ± 0.09 in MS + CJFJ2.5, 4.38 ± 0.15 in MS + CJFJ5 и 4.34 ± 0.15 in MS + CJFJ10). At the 30th minute, a significant increase in glucose in the MS group compared to the Control was observed (*p* < 0.05). CJFJ administration did not significantly affect glucose tolerance as compared to the MS group at any minute for the doses used.

### 2.4. Effects of Chaenomeles japonica Fruit Juice on Fat Indices

**Figure 3:**
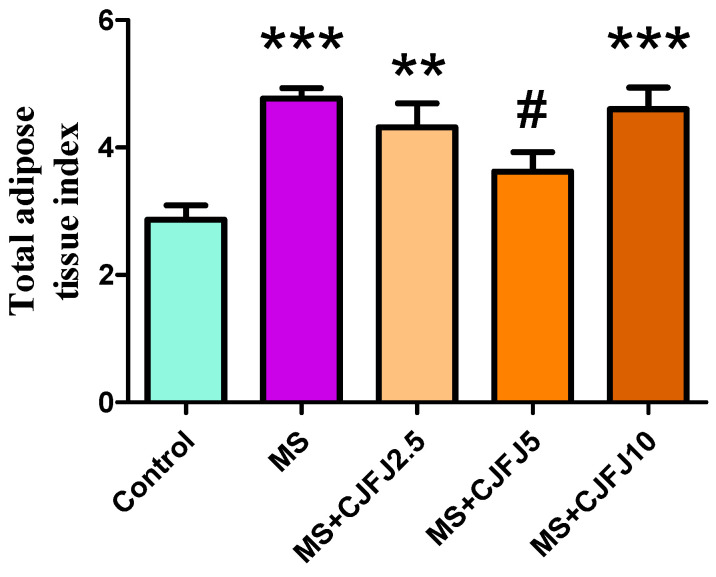
Total adipose tissue index in rats with diet-induced metabolic syndrome (MS), treated with *Chaenomeles japonica* fruit juice (CJFJ) at doses of 2.5 mL/kg, 5 mL/kg and 10 mL/kg. ** *p* < 0.01, *** *p* < 0.001 compared to group Control, # *p* < 0.05 compared to group MS.

**Figure 4:**
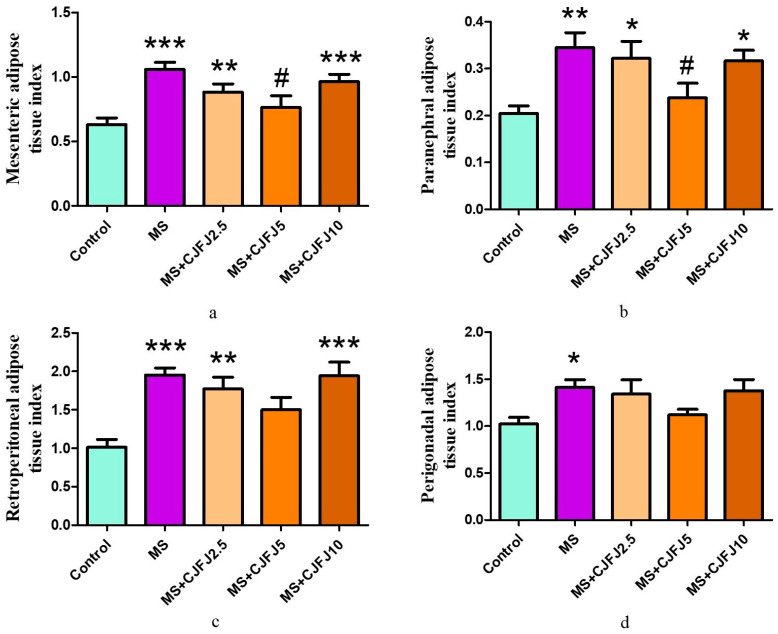
Mesenteric (**a**), paranephral (**b**), retroperitoneal (**c**) and perigonadal (**d**) adipose tissue indices in rats with diet-induced metabolic syndrome (MS), treated with *Chaenomeles japonica* fruit juice (CJFJ) at doses of 2.5 mL/kg, 5 mL/kg and 10 mL/kg; * *p* < 0.05, ** *p* < 0.01, *** *p* < 0.001 compared to Control group, # *p* < 0.05 compared to MS group.

The weights of the total, mesenteric, paranephral, perigonadal and retroperitoneal fat pads as well as their respective estimated indices were significantly increased in the MS group in comparison with the Control group. The total fat tissue index was significantly increased in groups MS, MS + CJFJ2.5 and MS + CJFJ10 compared to the Control, while its values in MS + CJFJ5 remained similar to those of the Control (Figure 3). A significant decrease in the total fat tissue index in the MS + CJFJ5 group compared to the MS group was observed. The CJFJ intake also affected (*p* = 0.0339) the mesenteric adipose tissue index in the MS + CJFJ5 group compared to the MS group (Figure 4a). The same dose of CJFJ also decreased the paranephral adipose tissue index in comparison to the MS group (Figure 4b).

### 2.5. Effects of Chaenomeles japonica Fruit Juice on Liver Index

**Figure 5:**
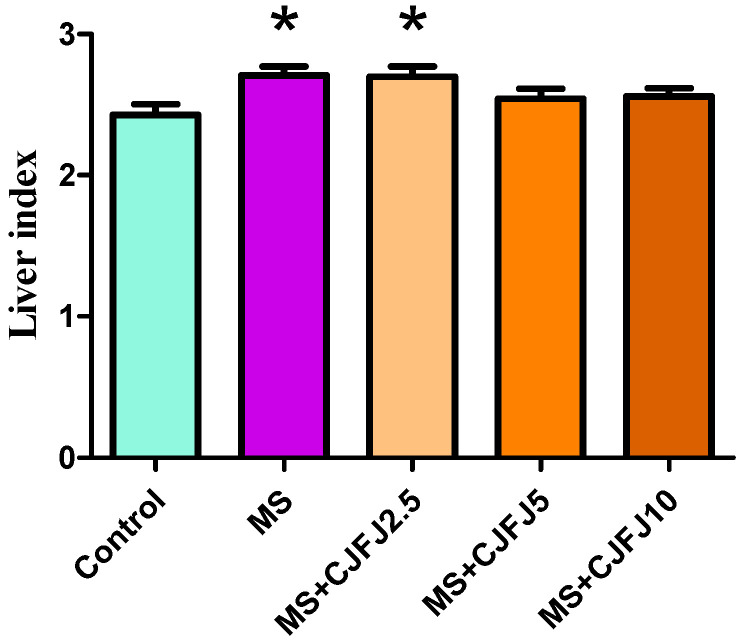
Liver index in rats with diet-induced metabolic syndrome (MS) treated with *Chaenomeles japonica* fruit juice (CJFJ) at doses of 2.5 mL/kg, 5 mL/kg and 10 mL/kg; * *p* < 0.05 compared to Control group.

The liver index results are presented in Figure 5. As a result of the HFHF diet, the liver index values were significantly increased in the MS and MS + CJFJ2.5 groups (*p* = 0.0343). The values observed in the MS + CJFJ5 and MS + CJFJ10 groups were comparable with those in the Control group.

### 2.6. Effects of Chaenomeles japonica Fruit Juice on the Lipid Profile

**Figure 6:**
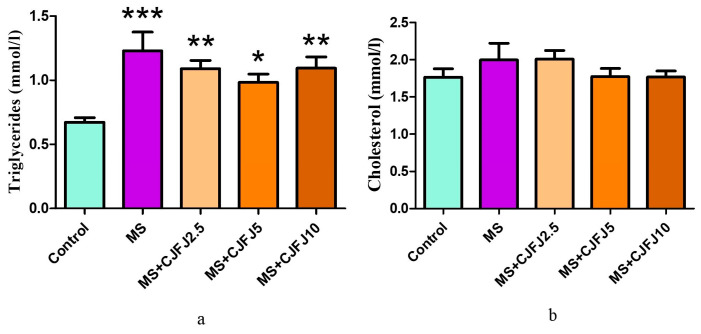
Serum triglycerides (**a**) and total cholesterol levels (**b**) in rats with diet-induced metabolic syndrome (MS) treated with *Chaenomeles japonica* fruit juice (CJFJ) at doses of 2.5 mL/kg, 5 mL/kg and 10 mL/kg; * *p* < 0.05, ** *p* < 0.01, *** *p* < 0.001 compared to Control group.

The serum triglyceride levels in the MS group were significantly increased (*p* < 0.001) in comparison with the Control group (1.23 ± 0.15 compared to 0.67 ± 0.04) (Figure 6a). Subjecting the animals to an HFHF diet led to almost doubling the values compared to the rats receiving the standard laboratory diet. The CJFJ intake reduced the triglyceride levels, but not significantly, and these changes were most pronounced in the MS + CJFJ5 group (0.98 ± 0.07 compared to 1.23 ± 0.15 in MS group). As shown in Figure 6b, the total cholesterol levels in the MS group were increased (2.00 ± 0.22) compared to the Control group (1.77 ± 0.11) without reaching statistical significance. CJFJ at any of the doses used did not significantly affect the total cholesterol levels.

### 2.7. Effects of Chaenomeles japonica Fruit Juice on Triglyceride/Glucose (TyG) Index

**Figure 7:**
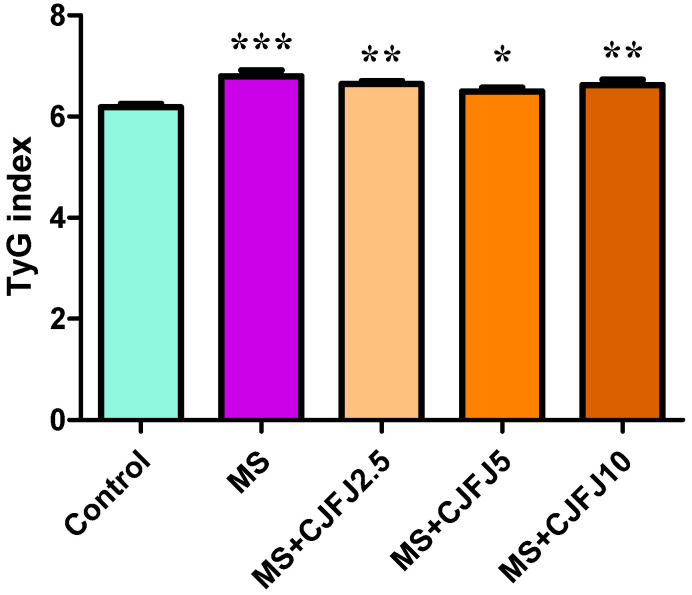
TyG index in rats with diet-induced metabolic syndrome (MS) treated with *Chaenomeles japonica* fruit juice (CJFJ) at doses of 2.5 mL/kg, 5 mL/kg and 10 mL/kg; * *p* < 0.05, ** *p* < 0.01, *** *p* < 0.001 compared to Control group.

The triglyceride/glucose (TyG) index was significantly increased in all groups of animals consuming an HFHF diet compared to the Control group (*p* = 0.0002), as observed in Figure 7. Administration of CJFJ did not significantly influence the TyG index.

### 2.8. Effects of Chaenomeles japonica Fruit Juice on Biochemical Markers of Antioxidant Defence and Oxidative Stress

**Figure 8:**
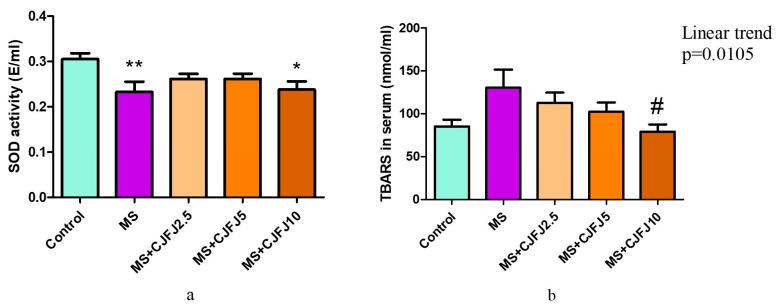
Activity of the enzyme superoxide dismutase (SOD) (**a**) and levels of thiobarbituric acid-reactive substances (TBARSs) (**b**) in the serum of rats with diet-induced metabolic syndrome (MS) treated with *Chaenomeles japonica* fruit juice (CJFJ) at doses of 2.5 mL/kg, 5 mL/kg and 10 mL/kg; * *p* < 0.05, ** *p* < 0.01 compared to Control group, # *p* < 0.05 compared to MS group.

The results from the serum activity of the antioxidant enzyme superoxide dismutase (SOD) are illustrated in Figure 8a. A statistically significant difference between the groups (*p* = 0.0173) was observed: there was lower activity of the enzyme in the MS group as well as in the MS + CJFJ10 group in comparison with the Control group. The treatment with CJFJ at doses of 2.5 mL/kg and 5 mL/kg prevented the HFHF diet-induced reduction in the enzyme activity, illustrated by the fact that there was no statistically significant difference between the Control group and the MS + CJFJ2.5 and MS + CJFJ5 groups.

In the serum of animals from group MS, higher values of thiobarbituric acid-reactive substances (TBARSs) were observed compared to the Control group (130.5 ± 21.09 versus 85.32 ± 7.73), but the difference did not reach statistical significance (Figure 8b). CJFJ intake caused a linear trend (*p* = 0.0105) towards a decrease in the TBARS serum levels. In the MS + CJFJ10 group, they were significantly reduced (*p* < 0.05) compared to those in the MS group, and they were comparable to the values of the Control group.

### 2.9. Correlations of SOD and TBARSs with Other Biochemical Parameters and with Fat Indices

**Figure 9:**
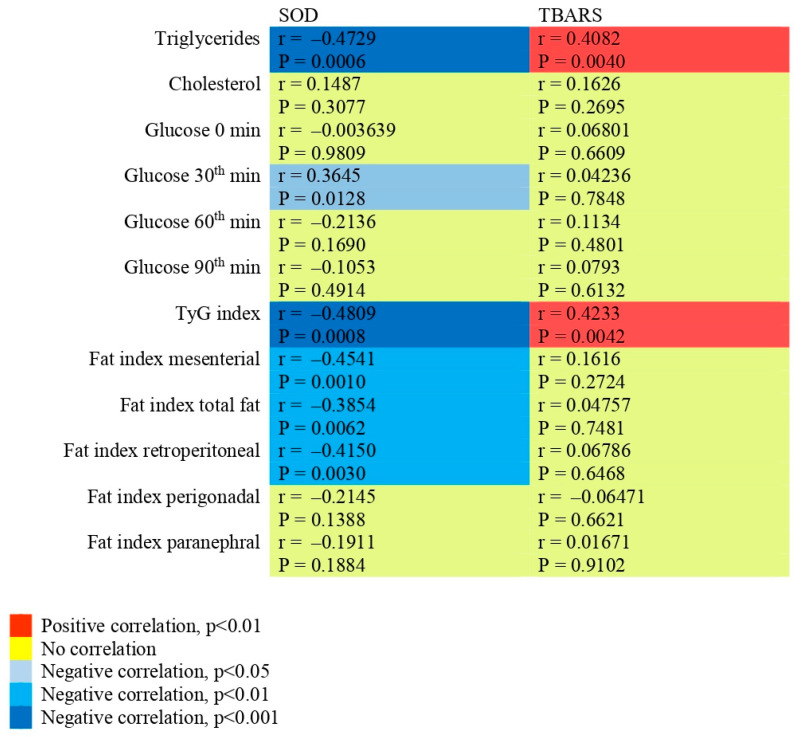
Pearson correlation heat map between SOD and TBARSs and other biochemical parameters and fat indices. The red colour represents a positive correlation and the blue colour represents a negative correlation. The depth of the colour stands for the size of the correlation coefficient. Darker colours visualize a stronger correlation, while lighter colours visualize a weaker correlation. The yellow colour represents no significant correlation.

As shown in Figure 9, SOD was negatively correlated with triglycerides (r = −0.473, *p* = 0.0006), the TyG index (r = −0.481, *p* = 0.0008), the mesenteric (r = −0.454, *p* = 0.0010) and retroperitoneal (r = −0.415, *p* = 0.0030) fat indices, and the glucose levels at the 30th min of GTT (r = −0.365, *p* = 0.0128). TBARSs were positively correlated with triglycerides (r = 0.408, *p* = 0.0040) and with the TyG index (r = 0.423, *p* = 0.0042). No correlation was found between both SOD and TBARSs and the rest of the parameters evaluated.

### 2.10. Effects of Chaenomeles japonica Fruit Juice on Liver and Adipose Tissue Histology

#### 2.10.1. Effects of *Chaenomeles japonica* Fruit Juice on Liver Histology

**Figure 10:**
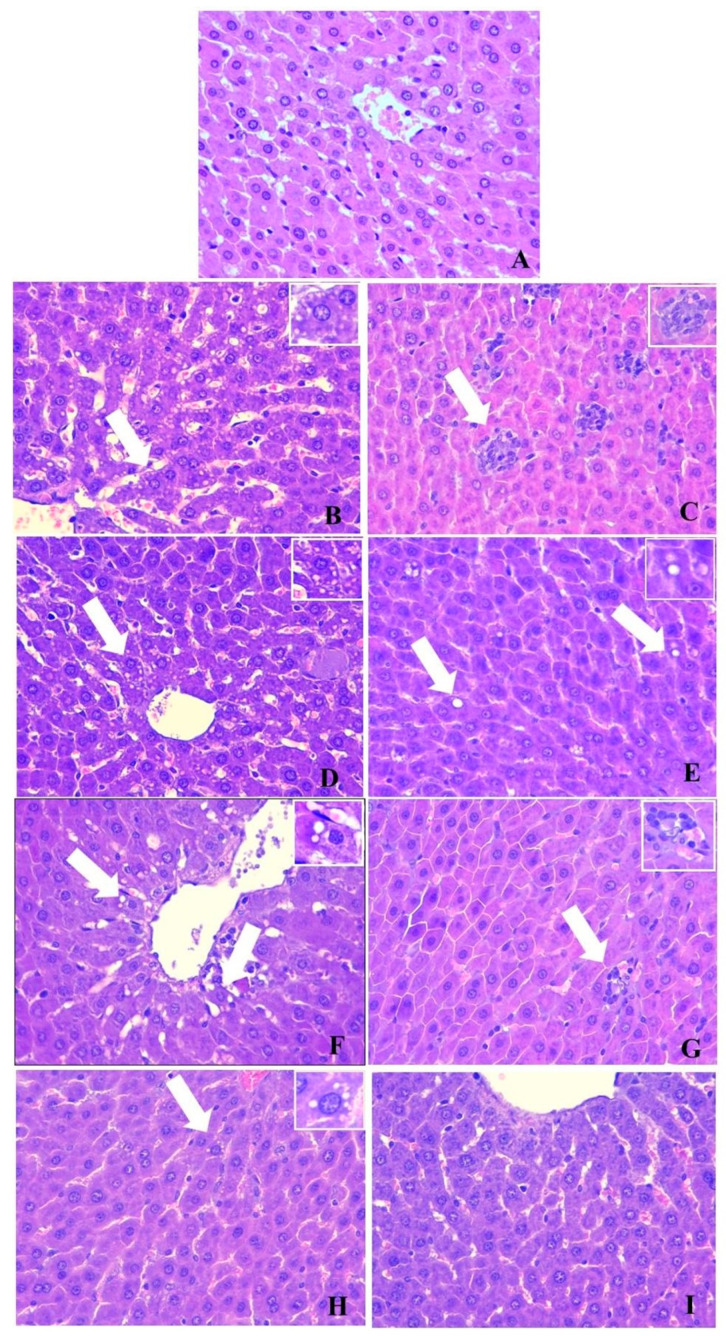
Microscopic appearance of the liver of the groups. Control (**A**): normal structure of liver tissue; MS: microvesicular steatosis around v. centralis—arrow (**B**), nonspecific granulomas—arrow (**C**); MS + CJFJ2.5: microvesicular steatosis around v. centralis—arrow (**D**), single hepatocytes with fatty degeneration—arrows (**E**); MS + CJFJ5: fatty degeneration around vena centralis—arrows (**F**), single nonspecific granulomas—arrow (**G**); MS + CJFJ10: single hepatocytes with fatty degeneration—arrow (**H**), normal structure of liver tissue (**I**); Hematoxylin–eosin staining, magnification ×200; Boxes—magnification × 400.

The results from the histopathological examination are presented in Figure 10. In the Control group, a normal structure of the liver was observed. In the animals from the MS group, a microvesicular steatosis, liver necrosis and non-specific granulomas were observed. In the MS + CJFJ2.5 group, a smaller number of hepatocytes were affected by the microvesicular steatosis. In the MS + CJFJ5 and MS + CJFJ10 groups, single non-specific granulomas and single hepatocytes with fatty degeneration were observed. Thus, the CJFJ treatment led to a dose-dependent reduction in the liver damage induced by the HFHF diet without completely preventing the occurrence of degenerative changes.

#### 2.10.2. Effects of *Chaenomeles japonica* Fruit Juice on Adipose Tissue Histology

**Figure 11:**
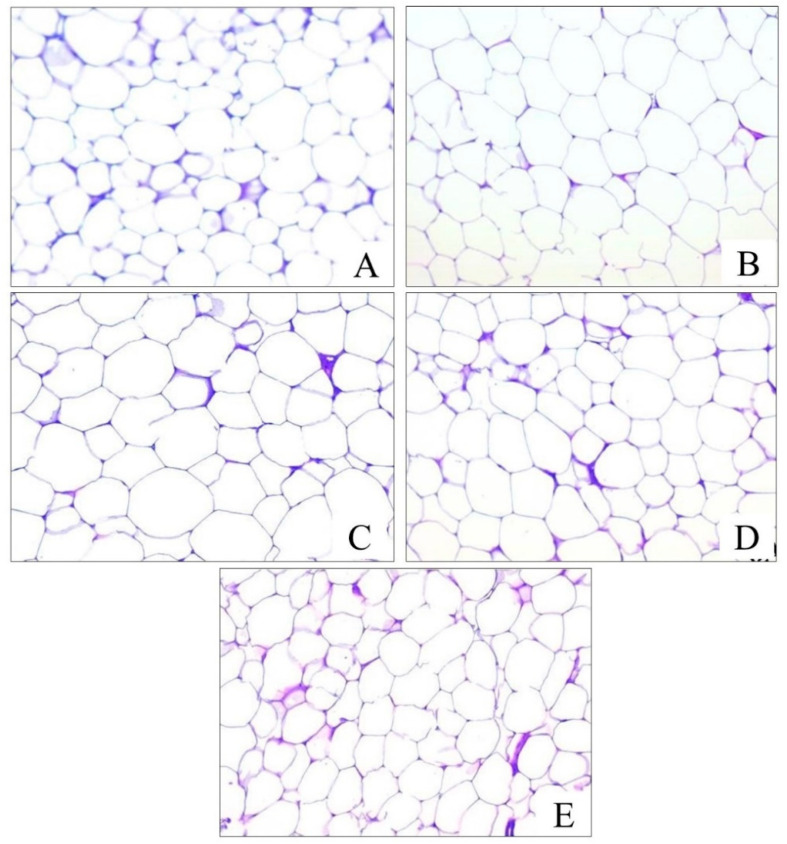
Histological structure of adipose tissue from groups: Control (**A**), MS (**B**), MS + CJFJ2.5 (**C**), MS + CJFJ5 (**D**) and MS + CJFJ10 (**E**); Hematoxylin–eosin staining, magnification × 200.

The microscopic appearance of the adipose tissue of the experimental animals is presented in Figure 11. The Control group demonstrated a normal structure of adipose tissue with small- and medium-sized adipocytes prevailing and single large-sized adipocytes also observed. In the animals from the MS group, adipocytes had a larger size compared to the Control group. Treatment with CJFJ dose-dependently prevented the development of HFHF-diet-induced adipocyte enlargement. In the MS + CJFJ2.5 group, large-sized adipocytes still prevailed, while in the MS + CJFJ5 and MS + CJFJ10 groups, the histological picture was similar to that observed in the Control group.

## 3. Discussion

All the animals on an HFHF diet consumed a smaller amount of food compared to the Control group. Probably, the elevated carbohydrate and lipid content of the HFHF diet contributed to a high energy density and caused a reduced food intake. What is more, part of the daily caloric intake was provided by the fructose solution. It has been continuously observed that calories ingested from fructose, especially in a liquid form, are not completely compensated for by reducing the amount of other food in order to maintain the usual daily caloric intake [17]. Instead, what is often observed is an increased energy consumption, which is consistent with our data—the energy intake was expectedly higher in all the animals consuming an HFHF diet. The liquid intake was higher in all groups fed an HFHF diet compared to the Control group. This is most probably due to the palatability of the fructose solution. The design of the current diet, most of all positioning fructose as the main carbohydrate, allows for the reproduction of the hedonistic features of the pathological feeding patterns that often contribute to the human MS condition. Additionally, fructose is known to induce thirst, possibly by increasing serum osmolality due to a shifting of water into the cell for the purpose of glycogenesis [18]. CJFJ at doses of 5 mL/kg and 10 mL/kg increased the amount of food consumed as well as the energy intake. This finding is consistent with one of the popular uses of *Chaenomeles* species in traditional medicine as an appetite stimulant [19]. According to the authors’ knowledge, this is the first scientific report in support of this traditional use. The liquid intake was reduced by CJFJ at a dose of 5 ml/kg. This is suggestive of a possible modulatory effect of the polyphenols found in CJFJ on fructose-induced thirst.

The body weight of the animals did not appear to be affected by the HFHF diet or by CJFJ. This is a common finding in diet-induced models of MS [20,21,22], and it resembles the clinical experience with humans, as visceral adiposity and not total body adiposity is a more accurate marker of MS [23]. In fact, distribution of body fat is considered a cornerstone in the pathogenesis of MS. Lipid accumulation in visceral depots, as well as in insulin-sensitive organs such as the liver and the skeletal muscles, plays a pivotal role in development and continuous reinforcement of insulin resistance as well as the typical low-grade inflammation [23,24]. At the same time, around 30% of obese people are metabolically normal, while 5–45% of people with a BMI in the reference range manifest the same metabolic disturbances that are typical of obese patients [24]. These are exactly the reasons why the body mass index is no longer considered the most appropriate way to estimate the obesity criteria as a part of MS; rather, the waist circumference or other markers are preferred for being more accurate. The increase in the total, mesenteric, paranephral, perigonadal and retroperitoneal fat tissue weights, as well as in their estimated indices, in the MS group in comparison to the Control group signifies the development of visceral obesity as a consequence of the HFHF diet. Treatment with CJFJ with the medium dose of 5 mL/kg led to a decrease in the total, mesenteric and paranephral fat tissue indices in comparison to the MS group, suggestive of anti-obesity properties. The loss of visceral fat tissue has been clinically verified to lead to a number of beneficial metabolic effects, including decreased systemic inflammation [25]; improvement of indices such as fasting blood glucose, triglycerides and the HOMA-index [26]; etc. A study with 172 obese adolescent participants considered visceral fat tissue reduction to be an independent predictor of ameliorating insulin resistance, hyperleptinemia and other metabolic disturbances [27]. Based on these data, the observed reduction in visceral adipose tissue by CJFJ at the medium dose of 5 mL/kg suggests that it could be a potentially appropriate diet intervention in patients with MS.

It is established that mesenteric fat, drained by the portal circulation, is metabolically more active than other nonportal adipose tissues; it was also found to be an independent determinant of MS and associated with an increased carotid intima-media thickness [28]. Mesenteric fat is considered a possible prognostic factor for fatty liver and polycystic ovary syndrome [29], with both conditions being deeply intertwined with MS. A significant correlation has been observed between mesenteric fat and both atherogenic LDL apoB particles and apoAII levels, with the latter being relevant to the role of mesenteric fat as a source of triglycerides in the fasting state [29]. While noticing anti-obesity properties in an appetite stimulant may seem somewhat surprising, such a combination is plausible if thermogenesis is affected or digestive enzymes (involved in the digestion of carbohydrates and fats) are inhibited. Thermogenesis in rodents is considered to contribute to 15–20% of daily energy expenditure [30]. Therefore, influencing this process can have a significant impact on the energy metabolism and body weight of animals. Several of the predominating polyphenols in CJFJ have been reported to have thermogenic properties: epicatechin, chlorogenic acid, ellagic acid [31], quercetin [32], vanillic acid [33], and p-coumaric [34,35]. At the same time, *Chaenomeles japonica* extract has already been shown to inhibit pancreatic lipase and α-amylase [36], so both of the aforementioned mechanisms could be involved in the anti-obesity properties we observed with the medium dose of 5 mL/kg CJFJ; however, in our experiment, we did not directly assess thermogenesis or digestive enzyme activity. This is a limitation of the study and a potential direction for future research. Interestingly, reduced visceral obesity was not observed at the highest dose of 10 mL/kg. The reason for this lack of response remains unclear and may reflect a non-linear nature of the observed dose–response relationship. One possible explanation is that the higher glucose and fructose content of the fruit juice might have counteracted the anti-obesity effects of its polyphenols.

Non-alcoholic fatty liver disease is considered the liver manifestation of MS. Nowadays, it is even referred to as metabolic dysfunction-associated fatty liver disease (MAFLD) [37]. It is a spectrum of pathological conditions, including steatosis, steatohepatitis, cirrhosis and hepatocellular carcinoma. Fructose-related stimulation of de novo lipogenesis and fatty liver has been observed in both animal and human studies [17]. The liver index values and histological analyses in this study suggest that CJFJ exerted a beneficial effect on the liver structure in diet-induced MS. On the subcellular level, increased endoplasmic reticulum stress is a key process involved in mediating the liver damage in MAFLD. The exceptional amounts of reactive oxygen species, initially originating from the food overload and later amplified by the many pathological pathways activated in MS, surpass the peroxisomes’ capacity to regulate them and spread to the endoplasmic reticulum where they alter the proper environment needed for protein folding. Chronic endoplasmic reticulum stress is associated with lipotoxicity, insulin resistance and inflammation [38]. Therefore, we can presume that the observed beneficial effects of CJFJ on liver histopahology are at least partly mediated by the antioxidant properties of the juice. Many of the polyphenols in CJFJ are reported to increase the liver expression of PPARα [39]—a transcriptional factor that is responsible for energy metabolism modulation and considered a possible target for MAFLD, MS and cardiovascular disease alleviation [40,41]. The effects seen in the liver in this experiment could also be related to the high carotenoid content of CJFJ [42]. Carotenoids express pronounced anti-inflammatory and antioxidant effects and modulate a number of intracellular pathways. They have been reported to inhibit steatosis by a change in inflammatory gene expression, regulation of the T-cell number and scavenging free radicals in similar experiments with rodents [43]. It should be noted that our study lacks data on liver enzyme activity as well as on the expression of genes and signalling pathways involved in hepatic lipid and carbohydrate metabolism and oxidative stress, which represents an important limitation of the present work in the context of evaluating the potential beneficial effects of CJFJ on the liver. Nevertheless, the primary focus of this study was the evaluation of changes in energy metabolism in the MS model. Future studies addressing these parameters would help to further clarify the mechanisms underlying the observed beneficial effects of CJFJ on the liver structure.

Data from the glucose tolerance tests suggested normoglycaemia, but impaired glucose tolerance as a result of the HFHF diet. CJFJ did not affect glucose tolerance in the doses used. While hypoglycaemic effects of *Chaenomeles japonica* polyphenolic extract have been reported in an in vitro study [44], according to our knowledge, this activity of the species has not yet been demonstrated in vivo.

Dyslipidemia is one of the central features of MS. It is a major risk factor for the development of atherosclerotic disease, ischemic heart disease, ischemic stroke, peripheral vascular disease, heart failure and sudden cardiac death [45]. Dyslipidemia develops partly in response to insulin resistance. The impairment of physiological insulin suppression of lipolysis in adipocytes results in increased levels of free fatty acids that act as a substrate for triglyceride synthesis in the liver [46]. Increased levels of oxidative stress and systemic inflammation also contribute to the development of dyslipidemia [47]. In this study, serum triglycerides were increased in all groups receiving the HFHF diet. This could be explained by the particular metabolic properties of fructose, a major component of the HFHF diet that is responsible for providing a significant part of the daily caloric intake of the experimental animals. It is known that fructose metabolism leads to the production of unregulated amounts of lipogenic substrates that are directly delivered to the mitochondria and that stimulate de novo lipogenesis in the liver, intrahepatic lipid accumulation and steatosis development, and hepatic insulin resistance [24]. As a response to fructose intake, long-lasting postprandial dyslipidemia is usually observed, ghrelin secretion is not adequately suppressed and leptin secretion is low, contributing to a continuous feeling of hunger despite the calories ingested [24,48,49]. The development of dyslipidemia in response to overconsumption of fructose has also been confirmed by clinical data [50]. In this study, no significant differences in the total cholesterol levels were observed among the experimental groups. These findings are consistent with the available literature showing largely unchanged total cholesterol levels resulting from low HDL cholesterol levels and increased LDL levels—key features of the atherogenic dyslipidemia found in MS [46]. In the present study, a measurement of individual lipoprotein fractions was not performed, which represents a limitation, as it would provide additional information on the metabolic effects of CJFJ. Despite lipid-lowering effects being reported for many of the polyphenols found in CJFJ [51], the triglyceride levels were not significantly affected in any of the doses used. Still, the triglyceride values in all CJFJ-treated groups remained lower than those in the MS group. This may be related to the fact that some of the effects of polyphenols in the body, such as modulation of gut microbiota—one of the mechanisms potentially involved in insulin resistance and dyslipidemia amelioration [52]—may require longer-term exposure in order to be manifested.

The TyG index has been recently used as a surrogate marker of insulin resistance [53]. In the present study, it was shown to be significantly increased in response to the HFHF diet, suggesting the development of insulin resistance. Treatment with CJFJ did not significantly affect the TyG index in the doses used, even though all estimated values in the treated groups were lower than those in the MS group.

Oxidative stress is not only a central feature of visceral obesity and MS but also one of the common denominators between MS and cardiovascular disease [54]. Decreased endogenous antioxidant abilities are a distinctive trait of MS and further contribute to aggravation of MS-induced tissue damage. SOD is a major endogenous free radical scavenger responsible for the breakdown of superoxide radicals [54]. The current study revealed a significant decrease in the serum SOD activity in rats with diet-induced MS, which is consistent with the existing literature [55]. The treatment with CJFJ at doses of 2.5 mL/kg and 5 mL/kg prevented the HFHF diet-induced reduction in SOD activity in the serum of animals, suggestive of improvement of the endogenous cellular antioxidant capacity. Restoration of physiological antioxidant capacities via serum SOD elevation was also shown for *Chaenomeles speciosa* fruit powder in an in vivo experiment with oxidative stress induced by exhaustive exercise in rats [56]. In this study, SOD was negatively correlated with triglycerides, the TyG index and the glucose levels on the 30th min of GTT, indicating a close relationship between oxidative stress and lipid and glucose metabolism. Such correlations have been previously observed [55].

The levels of thiobarbituric acid-reactive substances are evaluated as a marker of lipid peroxidation. In the current study, increased values were observed in the MS group compared to the Control group. Such findings have been described in rodent models of obesity as well as in humans with obesity and MS [57]. Serum levels of TBARSs have also been shown to be a strong and independent predictor of coronary artery disease [58]; they are further associated with the vascular event incidence, including fatal and non-fatal infarction and stroke [59]. CJFJ dose-dependently prevented lipid peroxidation. The levels of TBARSs in the MS + CJFJ10 group were comparable to those in the Control group. These results provide further evidence of the antioxidant properties of CJFJ.

Low-grade chronic inflammation is a hallmark of obesity and MS, contributing to insulin resistance and dysregulated lipid metabolism. Inflammatory markers were not measured in this study, which represents a limitation of the study and might be an object of further investigations.

In summary, CJFJ reduced visceral adiposity at the moderate tested dose and improved the oxidative status, which was associated with improved histopathological findings in the liver and adipose tissue. At the same time, the juice did not significantly affect systemic metabolic parameters such as glucose tolerance, insulin resistance, and triglyceride levels. A possible explanation for this discrepancy may lie in the fact that the root of the metabolic dysfunction—the increased fructose, lipid, and calorie intake—persisted until the end of the experiment.

## 4. Materials and Methods

### 4.1. Preparation and Storage of Chaenomeles japonica Fruit Juice

*Chaenomeles japonica* plants were grown in the Balkan mountains, Bulgaria, in the region of Troyan. The fresh fruits were handpicked, ground, crushed, and squeezed. The juice was filtered, preserved with potassium sorbate (1.0 g/L), and stored at 0 °C until the experiment.

### 4.2. Chemical Composition and Antioxidant Activity of Chaenomeles japonica Fruit Juice

**Figure 12:**
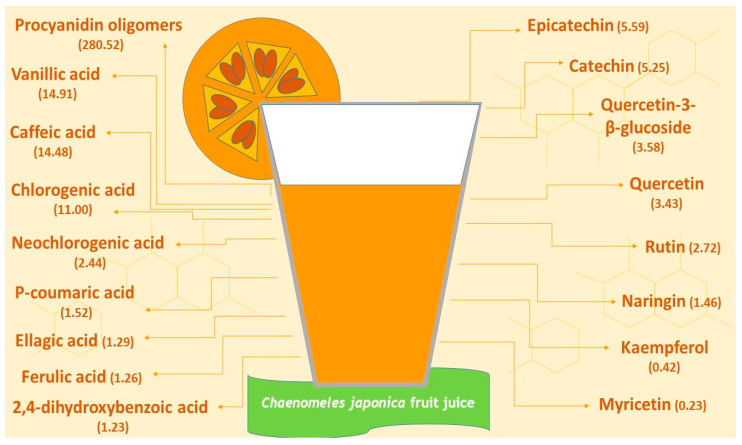
Polyphenolic content (mg/100 mL) of *Chaenomeles japonica* fruit juice as estimated by Valcheva-Kuzmanova et al., 2018 [60].

The chemical composition of CJFJ was determined by Valcheva-Kuzmanova et al., 2018 [60]. The total content of phenolic substances was spectrophotometrically estimated to be 890.00 mg GAE/L. High-performance liquid chromatography was performed and revealed a high procyanidin oligomer level and the presence of several phenolic acids and flavonoids. Among the flavonoids, epicatechin, catechin and quercetin-3-β-glucoside were most abundant (Figure 12). Among the phenolic acids, vanillic, caffeic and chlorogenic acid had the highest concentrations, followed by neochlorogenic, p-coumaric, ellagic, ferulic and 2,4-dihydroxybenzoic acid. Six organic acids, namely malic, quinic, citric, shikimic, ascorbic and oxalic acid, were also detected. Several carbohydrates were found in the juice: the predominant glucose (1713 mg/100 mL) and fructose (1237 mg/100 mL) as well as sucrose, xylose, rhamnose and arabinose. The antioxidant activity of CJFJ was estimated to be 18,167.8 ± 938.8 μmol gallic acid equivalents per litre by the hydroxyl radical averting capacity assay and 84,401.4 ± 1934.2 μmol Trollox equivalents per litre by the oxygen radical absorbance capacity assay [60].

### 4.3. Experimental Animals, Induction of MS and Treatment Protocol

Fifty adult male Wistar rats with an average initial weight of 270 ± 30 g bred in the Animal Centre of Medical University of Varna were used for the experiment. The animals were housed in plastic cages at a temperature of 22 ± 1 °C, in a well-ventilated room, with a 12-h light/dark cycle.

All procedures regarding the experimental animals were conducted in accordance with the European Union Directive 2010/63/EU for experiments with animals and approved by the Bulgarian food safety agency (document № 177/7 July 2017).

The rats were divided into five groups of ten animals each: Control, MS, MS + CJFJ2.5, MS + CJFJ5 and MS + CJFJ10. For ten weeks, rats from the Control group received a standard laboratory diet and tap water ad libitum. For the induction of MS, the other four groups were given an HFHF diet—17% lard and 17% fructose added to the standard diet as well as a 10% fructose solution instead of drinking water. Given the aforementioned diets, animals from the Control group received 279 kcal/100 g food while those on the HFHF diet received 405 kcal/100 g food and 40 kcal/100 mL fructose solution. While the standard diet provided 20.48 g of protein, 3 g of fat, 38.3 g of starch and 3 g of sugars per 100 g, the HFHF diet provided 13.65 g of protein, 18.67 g of fat, 25.5 g of starch and 19.55 g of sugars per 100 g. All animals were orally treated on a daily basis with a flexible orogastric tube. The Control and MS groups received distilled water (10 mL/kg), while the MS + CJFJ2.5, MS + CJFJ5 and MS + CJFJ10 groups were treated with CJFJ at increasing doses—2.5 mL/kg (diluted with distilled water to 10 mL/kg), 5 mL/kg (diluted with distilled water to 10 mL/kg), and 10 mL/kg, respectively. The doses were selected based on established guidelines for oral administration in rodents, with 10 mL/kg representing the generally accepted maximum volume for aqueous solutions when repeated dosing is intended [61]. Lower doses (5 and 2.5 mL/kg) were included to evaluate a potential dose–response relationship. Similar dosing regimens have been applied in our previous studies, where the biological effects of CJFJ were observed [62].

Food and liquid consumption was registered on a daily basis. The body weight of the animals was assessed once per week.

A glucose tolerance test was performed at the end of the 10th week.

At the end of the experiment, under ether anaesthesia, blood was collected from sublingual veins, centrifuged at 2000 r.p.m. for 10 min and stored at −20 °C until biochemical tests were performed.

After decapitation of the anesthetized animals, the fat deposits and livers were removed.

### 4.4. Glucose Tolerance Test (GTT)

After 12 h of fasting, the rats were intraperitoneally injected with glucose at a dose of 2 g/kg body weight. A blood sample from the distal end of the tail was collected. The blood glucose was measured with an ACCU-CHEK Performa glucometer by using ACCU-CHECK Performa test strips immediately before injection (0 min) as well as on the 30th, 60th and 90th minute after that. The results are presented in mmol/L.

### 4.5. Calculation of Tissue Indices

The liver and fat depot weights were expressed relative to the body weight rather than as absolute values to account for differences in body size between animals and to allow for meaningful comparisons between the experimental groups.

#### 4.5.1. Calculation of Fat Indices

Mesenteric, perigonadal, paranephral, and retroperitoneal fat depots were separately weighed. The total visceral adipose tissue was calculated. For each of the fat depots as well as for the total fat tissue, an index was measured using the following formula [63]:

$$Adipose\;tissue\;index=\frac{Adipose\;tissue\;weight}{Total\;body\;weight}\times 100$$

#### 4.5.2. Calculation of Liver Index

The liver index was determined using the following formula [64]:

$$Liver\;index=\frac{Liver\;weight}{Total\;body\;weight}\;\times \;100$$

### 4.6. Biochemical Measurements

#### 4.6.1. Estimation of Serum Triglycerides and Calculation of Triglyceride Glucose (TyG) Index

Triglycerides were measured in the serum by a colorimetric kit (Bio Maxima, Lublin, Poland) using the spectrophotometer AURIUS 2021 (Cecil Instruments Ltd., Cambridge, UK). The method was based on hydrolysis of triglycerides to glycerol and fatty acids by the enzyme lipoprotein lipase. As a result of the subsequent phosphorylation of glycerol with ATP by the glycerol kinase, glycerol-3-phosphate and ADP are produced. The glycerol-3-phosphate is oxidised to dihydroxyacetone phosphate and hydrogen peroxide that, upon binding to 4-chlorophenol and 4-aminoantipyrin, lead to the production of a coloured complex. The colour intensity was photometrically measured at a 500 nm wavelength.

The triglyceride glucose (TyG) index was determined in order to assess insulin resistance. The index was calculated using the formula of Lopez-Jaramillo et al., 2023 [53]:

$$TyG=Ln\frac{Fasting\;triglycerides\left(\frac{mg}{dl}\right)\times Fasting\;glucose(\frac{mg}{dl})}{2}$$

#### 4.6.2. Estimation of Serum Total Cholesterol

The total cholesterol levels were determined in the blood serum using the kits of Bio Maxima S.A., Poland, strictly following the instructions of the producer. The method was based on hydrolysis of cholesterol esters to cholesterol and free fatty acids by the enzyme cholesterol esterase. As a result of the subsequent oxidation of free cholesterol, hydrogen peroxide is released. It binds to phenol and 4-aminoantipyrin and leads to the production of a coloured complex. The colour intensity is photometrically measured at a 500 nm wavelength. The spectrophotometer AURIUS 2021 (Cecil Instruments Ltd., UK) was used.

#### 4.6.3. Superoxide Dismutase (SOD) Level Determination

An ELISA kit (Boster Bio, Pleasanton, CA, USA) was used for the superoxide dismutase level determination. The method was based on using a tetrazolium salt for the detection of a superoxide radical. The superoxide dismutase catalyses the dismutation of the superoxide anion to molecular oxygen and hydrogen peroxide. The concentration of the enzyme that is necessary for 50% dismutation of the superoxide radical is defined as one unit of superoxide dismutase. The results were read using the spectrophotometer AURIUS 2021 (Cecil Instruments Ltd., UK).

#### 4.6.4. Thiobarbituric Acid-Reactive Substance (TBARS) Level Determination

For the thiobarbituric acid-reactive substance level determination, a 0.8% thiobarbituric acid solution was added to the serum. The samples were incubated in a water bath at 95 °C for 2 h, and after that, were removed from the water bath and left to cool down at room temperature [65]. Malondialdehyde was used as a standard. The optical density of the samples was measured by an AURIUS 2021 spectrophotometer (Cecil Instruments Ltd., UK). The TBARSs were estimated as nmol/mL.

### 4.7. Histological Examination of Liver Tissue and Adipose Tissue

Pieces from the liver and from the retroperitoneal adipose tissue were fixed in 10% neutral buffered formalin and embedded in paraffin with a melting point of 52–54 °C in order to prepare the paraffin blocks. Sections with a 5 μm thickness were stained with hematoxylin–eosin (H and E). Light microscopy was used to assess the histological changes.

### 4.8. Statistical Analysis

A statistical analysis was performed using the GraphPad Prism 5 software. The results are presented as the mean ± SEM. A one-way analysis of variance (ANOVA) with Dunnet’s multiple comparison post-test was used. The correlations of SOD and TBARSs with other biochemical parameters and with the fat indices were tested by a correlation analysis using the Pearson test, two-tailed. Values of *p* < 0.05 were considered statistically significant.

## 5. Conclusions

The current study offers some insights into *Chaenomeles japonica* fruit juice intake in a model of metabolic syndrome. Although the fruit juice did not significantly affect the key parameters characteristic of metabolic syndrome, such as glucose intolerance, hypertriglyceridemia, and insulin resistance, several beneficial effects were observed. In particular, the treatment demonstrated dose-dependent antioxidant effects, improvement of histopathological changes in the liver and visceral adipose tissue, and reduction of visceral adiposity at the moderate tested dose. These findings suggest a potential supportive role of *Chaenomeles japonica* fruit juice in metabolic syndrome additionally to other measures aimed at improving the lipid profile and insulin resistance. However, further studies are needed to clarify the underlying mechanisms and to evaluate the potential therapeutic relevance.

## Figures and Tables

**Table 1:** Initial and final body weights in rats with diet-induced metabolic syndrome (MS) treated with *Chaenomeles japonica* fruit juice (CJFJ) at doses of 2.5 mL/kg, 5 mL/kg and 10 mL/kg.

	Control	MS	MS + CJFJ2.5	MS + CJFJ5	MS + CJFJ10
Initial body weight (g)	266.8 ± 8.08	262.3 ± 5.56	267.8 ± 5.56	267.2 ± 5.88	266.0 ± 5.43
Final body weight (g)	334.2 ± 8.21	344.0 ± 7.38	330.4 ± 9.00	336.0 ± 8.41	341.2 ± 10.61

**Table 2:** Plasma glucose levels during a glucose tolerance test, presented as absolute values (mmol/L) before as well as on the 30th, 60th and 90th min after the glucose load in rats with diet-induced metabolic syndrome (MS) treated with *Chaenomeles japonica* fruit juice (CJFJ) at doses of 2.5 mL/kg, 5 mL/kg and 10 mL/kg; * *p* < 0.05 compared to Control, ** *p* < 0.01 compared to Control, *** *p* < 0.001 compared to Control.

	Control	MS	MS + CJFJ2.5	MS + CJFJ5	MS + CJFJ10
0 min	4.46 ± 0.09	4.48 ± 0.14	4.44 ± 0.09	4.38 ± 0.15	4.34 ± 0.15
30th min	11.97 ± 0.78	15.92 ± 1.14 **	17.27 ± 0.72 ***	16.64 ± 1.26 **	15.58 ± 0.51 *
60th min	8.13 ± 0.26	9.93 ± 0.53	10.82 ± 0.74	9.83 ± 0.63	10.05 ± 0.56
90th min	7.41 ± 0.25	8.73 ± 0.28	8.95 ± 0.35	8.69 ± 1.07	8.34 ± 0.28

## Data Availability

The original contributions presented in this study are included in the article. Further inquiries can be directed to the corresponding author.

## Abbreviations

The following abbreviations are used in this manuscript:

MS	Metabolic syndrome
CJFJ	*Chaenomeles japonica* fruit juice
HFHF	High-fat high-fructose
FJ	Fruit juice
SOD	Superoxide dismutase
TBARSs	Thiobarbituric acid reactive substances
TyG	Triglyceride/glucose index
MAFLD	Metabolic dysfunction-associated fatty liver disease
